# Trajectory-guided dimensionality reduction for multi-sample single-cell RNA-seq data reveals biologically relevant sample-level heterogeneity

**DOI:** 10.1093/bioinformatics/btag192

**Published:** 2026-04-22

**Authors:** Haotian Zhuang, Xin Gai, Anru R Zhang, Wenpin Hou, Zhicheng Ji, Pixu Shi

**Affiliations:** Department of Biostatistics & Bioinformatics, Duke University, Durham, NC, 27710, United States; Department of Biostatistics, Vanderbilt University, Nashville, TN, 37232, United States; Department of Biostatistics & Bioinformatics, Duke University, Durham, NC, 27710, United States; Department of Computer Science, Duke University, Durham, NC, 27708, United States; Department of Biostatistics & Bioinformatics, Duke University, Durham, NC, 27710, United States; Department of Biostatistics, Columbia University, New York, NY, 10032, United States; Department of Biostatistics & Bioinformatics, Duke University, Durham, NC, 27710, United States; Department of Biostatistics & Bioinformatics, Duke University, Durham, NC, 27710, United States; Duke Microbiome Center, Duke University School of Medicine, Durham, NC 27710, United States

## Abstract

**Motivation:**

Dimensionality reduction for single-cell RNA-sequencing (scRNA-seq) data involving multiple biological samples presents a significant analytical challenge.

**Results:**

We introduce MUlti-Sample Trajectory-Assisted Reduction of Dimensions (MUSTARD), an innovative trajectory-guided dimensionality reduction method specifically designed for multi-sample, multi-condition scRNA-seq data. By integrating pseudotemporal information, MUSTARD provides a comprehensive unsupervised approach that simultaneously captures major gene expression variation patterns along pseudotime trajectories and across multiple samples, facilitating the discovery of biologically meaningful sample heterogeneity, endotypes, and associated gene markers and modules. In data-driven simulations, MUSTARD outperformed existing methods in distinguishing sample groups, achieving superior out-of-sample prediction accuracy. In two COVID-19 datasets and a tuberculosis dataset, MUSTARD identified components linked to symptom severity, batch effect, and other known biological variations, with notable overlap in immune response genes across the two independent COVID-19 datasets. These results underscore MUSTARD’s flexibility and power in identifying biologically relevant sample heterogeneity across diverse datasets.

**Availability and implementation:**

The R package MUSTARD with a detailed user manual is publicly available at https://github.com/haotian-zhuang/MUSTARD and Zenodo (DOI: 10.5281/zenodo.18293392). The source code to reproduce the results in this paper is available at https://github.com/haotian-zhuang/MUSTARD_Paper and Zenodo (DOI: 10.5281/zenodo.18293392).

## Introduction

The emergence of studies that collect multi-sample, multi-condition single-cell RNA-sequencing (scRNA-seq) data provides an unprecedented opportunity to explore the association between phenotype and cell-resolution transcriptomics. For example, several COVID-19 studies collected multiple scRNA-seq samples from patients with varied disease severities (Su et al. 2020, Stephenson et al. 2021, Tian et al. 2022). While many supervised methods have been proposed for cross-condition differential expression analysis (Crowell et al. 2020, Zhang et al. 2022), the options for unsupervised analysis of multi-sample scRNA-seq data are still limited. Dimensionality reduction is commonly used in single-cell analysis (Wolf et al. 2018, Stuart et al. 2019, Hao et al. 2021, Heumos et al. 2023). Many dimensionality reduction methods such as t-distributed Stochastic Neighbor Embedding (t-SNE) (Van der Maaten and Hinton 2008) and Uniform Manifold Approximation and Projection (UMAP) (Becht et al. 2018) have been developed or applied for scRNA-seq data from a single sample, aiming to extract cellular heterogeneity from noisy high-dimensional gene expression profiles by inferring a cell-level low-dimensional representation (Sun et al. 2019). However, there remains a paucity of methods for conducting dimensionality reduction on multi-sample scRNA-seq data, with several notable limitations in the existing literature. First, most existing methods aim to integrate multi-sample data (Stuart et al. 2019, Korsunsky et al. 2019), rather than identifying the driving factors behind sample-level heterogeneity. Second, majority of these methods can only infer a low-dimensional representation of cells, which can be difficult to connect to sample-level phenotypes. More importantly, current methods do not account for the pseudotemporal information within multi-sample data. Pseudotime analysis has been widely used to study the dynamics of biological processes by ordering cells along a pseudotime trajectory (Trapnell et al. 2014, Ji and Ji 2016, Qiu et al. 2017, Street et al. 2018). Early pseudotime methods, such as Monocle (Trapnell et al. 2014, Qiu et al. 2017), TSCAN (Ji and Ji 2016), and Slingshot (Street et al. 2018), ignore sample-to-sample variation and cannot identify changes in gene expression across conditions. A recent study demonstrated that genes exhibit multiple types of changes in a pseudotime trajectory across conditions and introduced Lamian, a regression framework for differential multi-sample pseudotime analysis (Hou et al. 2023). Leveraging such pseudotime trajectories provides a new path for the unsupervised analysis of multi-sample scRNA-seq data, including dimensionality reduction, enabling the connection between cell-level transcriptomics and sample-level heterogeneity such as phenotypes.

The existing literature on the dimensionality reduction for multi-sample scRNA-seq analysis remains limited. MEFISTO (Velten et al. 2022) and MOFAcell (Flores et al. 2023) are two such methods developed based on MOFA+ (Argelaguet et al. 2020). MEFISTO (Velten et al. 2022) can incorporate temporal information and infer a low-dimensional representation of cells. However, it does not generate a low-dimensional representation of samples, which is essential for capturing and establishing direct association with sample-level heterogeneity such as batch effects and phenotypes. MOFAcell (Flores et al. 2023) provides a low-dimensional representation of samples, but underutilizes single-cell resolution data by aggregating it into pseudobulk gene expression profiles. Similarly, a widely used approach in the literature (Caushi et al. 2021, Dykema et al. 2023), which we refer to as Pseudobulk-PCA, applies principal component analysis to pseudobulks formed by aggregating cells from the same sample, restricting its ability to leverage single-cell resolution. Table 1 summarizes the limitations of existing and related methods.

**Table 1:** Comparison of MUSTARD and other relevant methods. For each method, the columns indicate whether the method: (i) utilizes single-cell resolution, (ii) takes trajectory information into account, (iii) analyzes multiple samples, (iv) provides unsupervised analysis (does not require the sample-level covariate information), (v) infers a low-dimensional representation of samples instead of cells.

Method	Single-cell resolution	Trajectory information	Multi-sample	Unsupervised	Sample-level dimensionality reduction
MUSTARD	Yes	Yes	Yes	Yes	Yes
Monocle	Yes	Yes	No	No	No
TSCAN	Yes	Yes	No	No	No
Slingshot	Yes	Yes	No	No	No
IDEAS	Yes	No	Yes	No	No
Lamian	Yes	Yes	Yes	No	No
MOFA+	Yes	No	Yes	Yes	No
MEFISTO	Yes	Yes	Yes	Yes	No
MOFAcell	No	No	Yes	Yes	Yes
Pseudobulk-PCA	No	No	Yes	Yes	Yes

**Figure 1:**
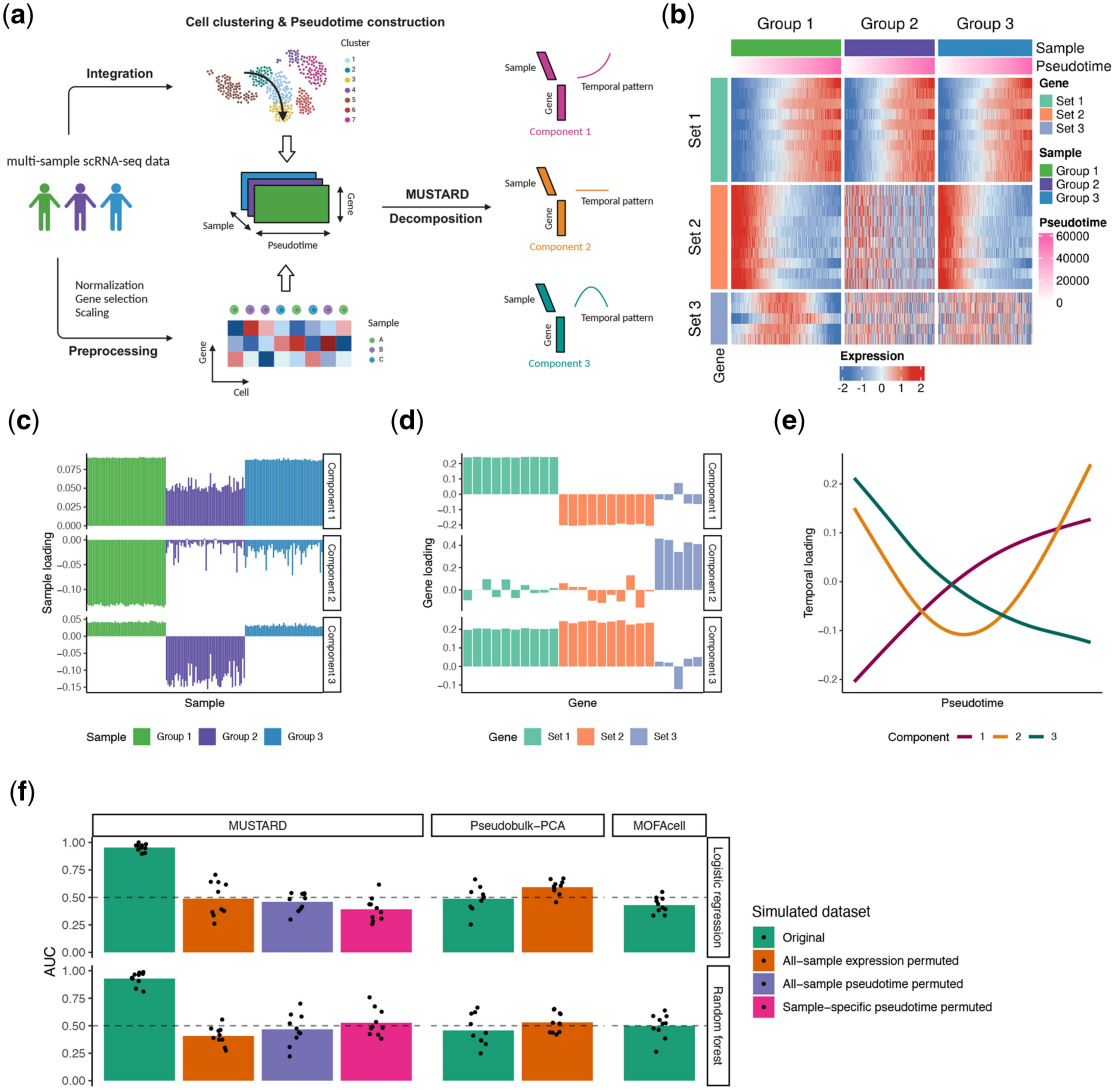
Overview of MUSTARD with simulation studies. (a) A schematic view of MUSTARD. (b) Heatmap showing the multi-sample pseudotemporal expression patterns in the simulated dataset. (c-e) Sample loadings (c), gene loadings (d), and temporal loadings (e) of the top three MUSTARD components from the simulated dataset. The first component captures the monotone trend of genes in Sets 1 and 2, with negative gene loading for the decreasing trend in Set 2, and smaller sample loading for Group 2 due to no trend is in Set 2. The second component captures the U-shaped trend of genes in Set 3 from Group 1. The third component supplements the first component. (f) Performance evaluation of MUSTARD, Pseudobulk-PCA, and MOFAcell using the simulated datasets (for details see Methods). Note that for Pseudobulk-PCA and MOFAcell, the all-sample pseudotime permuted and sample-specific pseudotime permuted datasets are equivalent to the original dataset. Note that for the null scenarios, MOFAcell gave all zero loadings for all components, making the predictive modeling infeasible.

To this end, we present MUlti-Sample Trajectory-Assisted Reduction of Dimensions (MUSTARD), a trajectory-guided method for the dimensionality reduction of multi-sample scRNA-seq data. MUSTARD is a pioneering method that utilizes single-cell resolution information to provide unsupervised low-dimensional representation of samples while simultaneously connecting the sample-level heterogeneity with gene modules and pseudotemporal patterns. MUSTARD requires three inputs: a gene expression matrix for all cells, a categorical vector indicating which sample each cell belongs to, and the pseudotime values for each cell constructed based on the multi-sample scRNA-seq data using any method appointed by the user according to their cell types and trajectories of interest. After standard preprocessing steps (e.g. feature filtering and scaling), we format the data into an order-3 temporal tensor with sample, gene, and pseudotime as its three dimensions. The tensor is then decomposed into the summation of low-dimensional components, where each consists of a sample loading vector, a gene loading vector, and a temporal loading function (Fig. 1a). This all-in-one decomposition is capable of simultaneously revealing sample heterogeneity, major gene expression variation patterns and gene pathway enrichment along the trajectory, thus facilitating sample-level analysis of single-cell data and providing opportunities to discover sample endotypes and corresponding genetic signatures.

## Materials and methods

### MUSTARD algorithm

#### Pseudotime input

MUSTARD takes as input a user-provided one-dimensional pseudotemporal coordinate for each cell (e.g. pseudotime). More generally, the input can be any one-dimensional numeric ordering of interest, provided the values are comparable across samples. In practice, when pseudotime trajectories are constructed, we recommend first integrating cells from multiple samples into a shared low-dimensional space using methods such as Seurat (Stuart et al. 2019), Harmony (Korsunsky et al. 2019), or scVI (Lopez et al. 2018), followed by trajectory inference in this common space. MUSTARD operates under the assumption that the pseudotime of the cells is comparable across samples.

#### Tensor decomposition

To connect sample-level heterogeneity to gene programs and their pseudotime dynamics while retaining single-cell resolution information, we represent multi-sample scRNA-seq data as a sample by gene by pseudotime tensor and decompose it into components with sample, gene, and temporal loadings. The sample loadings provide a low-dimensional embedding of samples that summarizes sample-level heterogeneity, whereas the gene loadings and temporal loadings respectively identify the genes driving each component and their pseudotemporal expression patterns underlying that heterogeneity. MUSTARD itself operates on the original (non-integrated) gene expression profiles to preserve sample-level heterogeneity in its input.

Specifically, denote Y as the order-3 temporal tensor after the preprocessing steps (e.g. library-size-normalization and log-transformation to make the data more Gaussian-like), with *m* samples and *n* genes, and $y_{\mathrm{ijt}}$ as the expression level of gene *j* from sample *i* at pseudotime $t\in T_{i}$ which can be different across samples.

The core of the MUSTARD algorithm is to decompose Y using an approximately CP low-rank structure (Han et al. 2024):


$$y_{\mathrm{ijt}}=\sum_{\ell =1}^{r}\lambda^{\left(l\right)}a_{i}^{\left(l\right)}b_{j}^{\left(l\right)}\xi^{\left(l\right)}(t)+\epsilon_{\mathrm{ijt}},$$

where *r* is the number of low-rank components to approximate Y, $\epsilon_{\mathrm{ijt}}$ represents measurement error, $a^{\left(l\right)}=(a_{1}^{\left(l\right)},\ldots ,a_{m}^{\left(l\right)})$, $b^{\left(l\right)}=(b_{1}^{\left(l\right)},\ldots ,b_{n}^{\left(l\right)})$, and $\xi^{\left(l\right)}$ are the sample loading, gene loading, and temporal loading for component *l*, respectively. We estimate the components sequentially using a deflation scheme by minimizing the following objective function one component at a time for ℓ=1,…,r,


$$\sum_{i=1}^{m}\sum_{j=1}^{n}\sum_{t\in T_{i}}{\left\{y_{\mathrm{ijt}}-\lambda^{\left(l\right)}a_{i}^{\left(l\right)}b_{j}^{\left(l\right)}\xi^{\left(l\right)}(t)\right\}}^{2}+C_{K}||\xi^{\left(l\right)}|{|}_{H}^{2}$$

subject to the following constraints:


$$\sum_{i=1}^{m}{\left[a_{i}^{\left(l\right)}\right]}^{2}=\sum_{j=1}^{n}{\left[b_{j}^{\left(l\right)}\right]}^{2}=\int {\left[\xi^{\left(l\right)}(t)\right]}^{2}dt=1,$$

with Y updated at each ℓ by subtracting the previously estimated components (for details see Supplementary Materials). Here, $\lambda =(\lambda^{\left(1\right)},\ldots ,\lambda^{\left(r\right)})$ quantifies the contribution of each component. $||\xi |{|}_{H}$ is the reproducing kernel Hilbert space (RKHS) norm of function ξ(t) with the rescaled Bernoulli polynomial kernel


$$K(s,t)=1+k_{1}(s)k_{1}(t)+k_{2}(s)k_{2}(t)-k_{4}(|s-t|)$$

where $k_{1}(s)=s-0.5$, $k_{2}(s)=[k_{1}^{2}(s)-1/12]/2$, and $k_{4}(s)=[k_{1}^{4}(s)-k_{1}^{2}(s)/2+7/240]/24$. Explicitly,


$$\begin{array}{cc} ∥\xi {∥}_{H}^{2}=\int_{0}^{1}{\left(\xi^{\prime \prime }(t)\right)}^{2}dt+{\left(\int_{0}^{1}\xi (t)dt\right)}^{2}+{\left(\int_{0}^{1}\xi (t)(t-\frac{1}{2})dt\right)}^{2} & \\ +{\left(\int_{0}^{1}\xi (t)\frac{{\left(t-\frac{1}{2}\right)}^{2}-\frac{1}{12}}{2}dt\right)}^{2}. & \end{array}$$

Therefore, by choosing the Bernoulli polynomial kernel above, the RKHS norm imposes a penalty on the squared second derivative of ξ, together with additional quadratic terms that control its projections onto the polynomial basis of order up to two $\left\{1,t-1/2,{\left(t-1/2\right)}^{2}\right\}$. Such a setting penalizes undesirable function behaviors and enforces smoothness in the inferred functions ξ(t) over pseudotime *t*, thus mitigating the impact of noisy fluctuations commonly observed in single-cell data. Consequently, the learned trajectories show gradual and interpretable trends in gene expression dynamics, which are more likely to reflect genuine biological processes such as developmental programs, rather than spurious variation.

A tuning parameter $C_{K}$ controls the smoothness of ξ(t). This model setting and the corresponding decomposition algorithm are supported by established statistical theory (Han et al. 2024) and have been successfully applied to microbiome data (Shi et al. 2024). For convenience, we provide detailed descriptions of the algorithm, including the selection of rank *r*, in the Supplementary Materials.

#### Sample phenotype prediction

Denote $Y_{\text{train}}$ and $Y_{\text{test}}$ as the temporal tensor from the training samples and testing samples, respectively, where the trajectory was defined on all cells. For each component l=1,…,r of $Y_{\text{train}}$, denote $a_{\text{train}}^{\left(l\right)}$, $b_{\text{train}}^{\left(l\right)}$, and $\xi_{\text{train}}^{\left(l\right)}$ as its sample loading, gene loading, and temporal loading, respectively. Assuming $Y_{\text{train}}$ and $Y_{\text{test}}$ share the same gene loading and temporal loading, we can estimate the sample loading of $Y_{\text{test}}$ (i.e. $a_{\text{test}}^{\left(l\right)}$) by plugging $b_{\text{train}}^{\left(l\right)}$ and $\xi_{\text{train}}^{\left(l\right)}$ in Step 2 of the MUSTARD algorithm (Supplementary Materials). As a result, any predictive model (e.g. logistic regression, random forest) trained on the training data’s sample loading $a_{\text{train}}^{\left(l\right)}$ and phenotype can be transferred to $a_{\text{test}}^{\left(l\right)}$ to predict the phenotype of the testing sample.

Logistic regression was implemented using the R function glm() from the R package stats (version 4.2.2). For multi-group prediction (≥3), multinomial logistic regression was implemented using the R function multinom() from the R package nnet (version 7.3.18). Random forest was implemented using the R package randomForest (version 4.7.1). AUC-ROC was calculated using the R package pROC (version 1.18.4).

#### Gene module identification and representation

Given $\lambda =(\lambda^{\left(1\right)},\ldots ,\lambda^{\left(r\right)})$ and $b=(b^{\left(1\right)},\ldots ,b^{\left(r\right)})$, we denote $B=(B^{\left(1\right)},\ldots ,B^{\left(r\right)})$ as the gene embedding matrix, where $B^{\left(l\right)}=\lambda^{\left(l\right)}b^{\left(l\right)}$ and l=1,…,r. For each low-dimensional component *l* identified by the tensor decomposition, the corresponding gene embedding score $B^{\left(l\right)}$ reflects the contribution of each gene. In general, genes with similar pseudotemporal patterns to the temporal loading $\xi^{\left(l\right)}$ have larger embedding scores, although some genes may combine pseudotemporal patterns from multiple components. Therefore, these gene embedding scores can be used to cluster genes into modules with similar pseudotemporal patterns. Users have the option to use any clustering method on the gene embedding matrix with preferred distance measures. In this study, hierarchical clustering with Pearson correlation distance measure was implemented using the R function hclust(method = “average”) from the R package stats (version 4.2.2). UMAP was implemented using the R function umap(metric = “correlation”) from the R package uwot (version 0.2.1). GO enrichment was performed using the R package topGO (version 2.50.0). The R package ComplexHeatmap (version 2.15.4) was used for visualization. The expression of genes from the same module were averaged to form a metagene.

#### Cross-study overlap proportion

**Figure 2:**
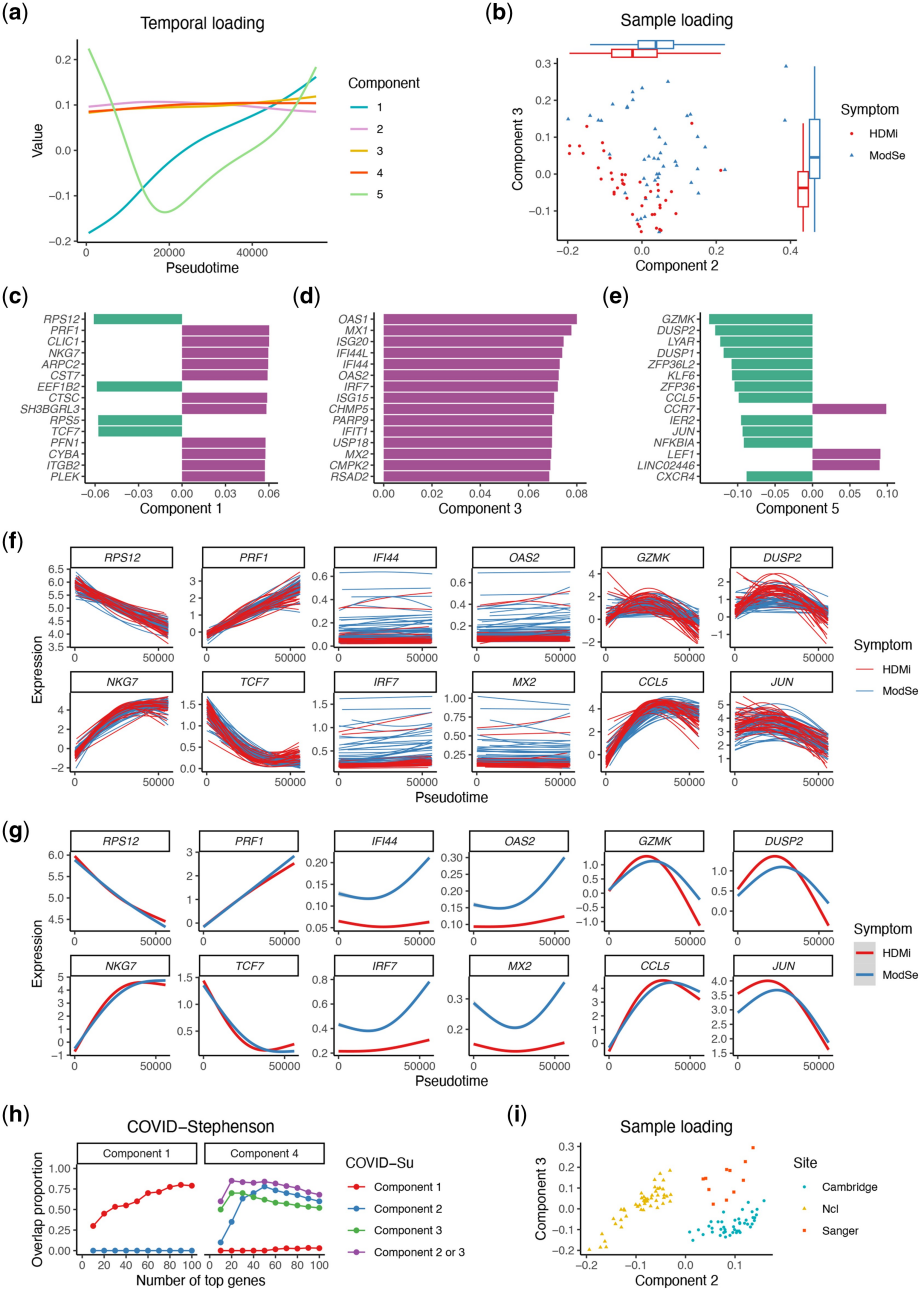
Analysis of COVID-19 studies. (a-g) Results of the COVID-Su study. (a) Temporal loadings capture major temporal patterns. (b) Component 2 and 3 jointly separate samples from different severity levels. *P*-values obtained by Wilcoxon rank-sum test are $2.04\times 10^{-3}$ and $1.78\times 10^{-5}$, respectively. (c-e) Top genes in Component 1 (c), Component 3 (d), and Component 5 (e). (f-g) Example genes’ temporal patterns for each sample (f) and each group (g). (h) Proportion of overlapping genes among top *L* genes between the two COVID-19 studies for a range of *L* (for details see Methods), where components depicting the same process sharing more overlap between studies. (**i**), In the COVID-Stephenson study, Component 2 separates samples from Ncl, and Component 3 further separates samples from Cambridge and Sanger.

To quantify the consistency of our findings in genes across two studies, we calculate the proportion of top-ranked genes identified by each component that are overlapped between studies. Specifically, denote *A* and *B* as the set of top *L* genes identified in two studies. |A|=|B|=L, where |.| is the cardinality of a set. The overlap proportion is defined as $\frac{\left|A\cap B\right|}{L}$, where *L* is selected to be a range of values (for an example see Fig. 2h). The overlap proportion is expected to be higher when comparing components with similar pseudotime patterns, and lower when comparing unrelated components.

### Competing methods

To obtain sample-level dimensionality reduction from scRNA-seq data, a common approach in the literature (Caushi et al. 2021, Dykema et al. 2023) is through the principal component analysis of pseudobulks formed by aggregating cells from the same sample, a method we denoted as the Pseudobulk-PCA method. Specifically, when implementing Pseudobulk-PCA in the benchmarking, we took the average expression across all cells for each sample, and standardized each gene’s expression to have zero mean and unit variance. PCA was then performed to extract the top principal components as low-dimensional representation of the samples. MOFAcell was implemented using the R packages MOFA2 (version 1.13.0) and MOFAcellulaR (version 0.0.0.9000).

### Data preprocessing

Before the MUSTARD analysis, users first need to specify and provide the pseudotime trajectory they aim to analyze, which can be any one-dimensional numeric values of interest. The majority of existing methods for the construction of pseudotime trajectories require the integration of multi-sample scRNA-seq data beforehand (Stuart et al. 2019, Korsunsky et al. 2019). However, the corrected expression values from integration methods, whose biological variation across samples are removed, will not be used by MUSTARD. Instead, the original gene expression profiles should go through standard preprocessing steps, including normalization and identification of highly variable genes (HVGs) before being used as input for MUSTARD. Since our method uses a tensor decomposition designed for continuous data, data imputation [e.g. SAVER (Huang et al. 2018)] is also recommended to mitigate the zero values in the expression before log-transformation and scaling. The following sections describe in detail the preprocessing steps we employed for the datasets used in this paper.

#### COVID-Su dataset

The COVID-Su dataset (Su et al. 2020) consisting of 270 samples from 129 COVID-19 patients and 16 healthy donors was downloaded from the ArrayExpress website https://www.ebi.ac.uk/biostudies/arrayexpress/studies/E-MTAB-9357. SAVER (Huang et al. 2018) (version 1.1.2) was used to impute the library-size-normalized count data from each sample, followed by log2-transformation. Consistent with a previous study (Hou et al. 2023), CD8+ T cells were annotated based on the SAVER-imputed values of *CD3D* and *CD8A*. Cells with fewer than 500 expressed genes, fewer than 2000 counts, or more than 10% mitochondrial counts were filtered out. We retained 161 samples with more than 500 cells and 100 CD8+ T cells. Seurat (Stuart et al. 2019) (version 3.2.1) was used to integrate data from multiple samples and TSCAN (Ji and Ji 2016) (version 1.7.0) was applied to construct a pseudotime trajectory using 55 953 naive and CD8+ T cells with default settings. The trajectory was divided into 50 consecutive intervals (bins) of equal length because we found that 50 bins provided enough resolution to characterize the pseudotime trajectory of the cells in this dataset, and more bins did not lead to meaningful changes in the results. For a given gene and a given sample, the expression value within each bin was represented by the median of the log2-transformed SAVER-imputed expression values in cells with pseudotime falling into the bin, and the corresponding pseudotime of the binned cell was represented by the midpoint of the bin. After cell binning, genes with expression values higher than 0.1 in at least 5% of cells were retained. We next applied a gene-specific local polynomial regression (LOESS) to fit the relationship between the standard deviation and the mean of expression values. Genes with positive residuals were selected as HVGs, and then standardized to have zero mean and unit variance. Because a proportion of patients have repeated sampling, we only focused on the 89 samples collected at the first time point from participants, consisting of 28 mild, 27 moderate, and 19 severe COVID-19 patients, and 15 healthy donors. After preprocessing, a final set of 1600 genes on 3719 metacells was formatted into an order-3 temporal tensor with 89 samples, 1600 genes, and 50 pseudotime values as its three modes. Since previous studies have shown a high consistency between healthy to mild samples, and between moderate to severe samples (Su et al. 2020), we dichotomized the severity levels into two groups, i.e. healthy or mild (HDMi) and moderate or severe (ModSe).

#### COVID-Stephenson dataset

The COVID-Stephenson dataset (Stephenson et al. 2021) consisting of 143 samples from three centers (Ncl, Cambridge, and Sanger) was downloaded from the ArrayExpress website https://www.ebi.ac.uk/biostudies/arrayexpress/studies/E-MTAB-10026. Similar to the COVID-Su dataset, we only focus on the 110 samples collected at the first time point from 9 mild, 53 moderate and 24 severe COVID-19 patients, and 24 healthy donors. These 110 samples comprised 51 from Ncl, 48 from Cambridge, and 11 from Sanger. The severity of COVID-19 in each sample was standardized based on the World Health Organization (WHO) ordinal score, which was used in the COVID-Su dataset. We followed the same processing steps as described in the COVID-Su dataset, except that the pseudotime trajectory was constructed using Harmony-integrated low-dimensional embeddings and cell type annotations obtained from the original data. After feature selection and scaling, a final set of 1833 genes on 4614 metacells was formatted into an order-3 temporal tensor with 110 samples, 1833 genes, and 50 pseudotime values as its three modes.

#### Tuberculosis (TB) dataset

The TB dataset (Nathan et al. 2021) consisting of 500 089 memory T cells from 259 individuals was downloaded from the GEO website https://www.ncbi.nlm.nih.gov/geo/query/acc.cgi?acc=GSE158769. We retained 184 samples from 84 males and 100 females with more than 1000 cells, using the same filtering criteria as in a recent study (Hou et al. 2023). A pseudotime trajectory reflecting the T cell activation process was directly obtained from the authors of Lamian (Hou et al. 2023). After the same processing steps, a final set of 1790 genes on 9038 metacells was formatted into an order-3 temporal tensor with 184 samples, 1790 genes, and 50 pseudotime values as its three modes.

#### Simulated data

To generate a realistic dataset with multiple samples and different pseudotemporal patterns, we first manually selected a subset of genes with clear pseudotemporal patterns in the COVID-Su dataset. To be specific, we selected 10 genes with increasing trend (Set 1), 10 genes with decreasing trend (Set 2), and 5 genes with a single peak (Set 3) across all samples. The gene expression is fitted along pseudotime by the generalized additive model (GAM). GAM was implemented using the R function gam() from the R package mgcv (version 1.8.41) with the formula y∼s(t,k=3), same as TSCAN (Ji and Ji 2016). Next, we randomly divided 161 samples into three groups (Groups 1–3). For genes in Set 2, we permuted the expression within each sample in Group 2. For genes in Set 3, we permuted the expression within each sample in Group 2 and Group 3. Finally, a Gaussian noise with zero mean and standard deviation of 0.1 was added back to the fitted expression values. To evaluate the performance of sample phenotype prediction, the standard deviation of the Gaussian noise was increased to 3. We performed the same binning and scaling steps as described above. The top three MUSTARD components were used for downstream analyses.

Building upon the simulated dataset above, we then created three null scenarios using different permutation strategies (all-sample expression permuted, all-sample pseudotime permuted, and sample-specific pseudotime permuted) that break the connection between sample-level differences and cell-level transcriptomics. For all-sample expression permuted dataset, the expression of each gene was permuted across all cells from all samples. For all-sample pseudotime permuted dataset, the pseudotime of cells was permuted across all samples but the cell-sample connection was preserved. For sample-specific pseudotime permuted dataset, the pseudotime of cells was permuted within each sample. Note that when using the Pseudobulk-PCA method and MOFAcell, the all-sample pseudotime permuted and sample-specific pseudotime permuted datasets are equivalent to the original dataset.

## Results

First, we demonstrate the capability of MUSTARD through a data-driven simulation based on the COVID-Su dataset (Su et al. 2020). This dataset was chosen because a previous study (Hou et al. 2023) has constructed a pseudotime trajectory from naive T cells to CD8+ T cells and identified genes with significant expression trends along the trajectory. We focused on genes with three different pseudotemporal patterns (Sets 1–3), randomly divided samples into three groups (Groups 1–3), and randomly shuffled the expression of certain genes within each sample in certain groups to differentiate the sample groups (Fig. 1b, for details see Methods). Figure 1c-e illustrated the result of MUSTARD for one simulation round, decomposes the simulated multi-sample pseudotemporal data into informative components that depict the differences between sample groups (Fig. 1c) and between gene sets (Fig. 1d) in terms of their pseudotemporal trends (Fig. 1e). We compared MUSTARD with MOFAcell and Pseudobulk-PCA (for details see Methods), both of which were unable to distinguish the sample groups as illustrated in Fig. 1, available as supplementary data at *Bioinformatics* online for one simulation round.

To further quantify the superiority of MUSTARD over Pseudobulk-PCA and MOFAcell in their ability to capture sample heterogeneity, sample loadings of the top three components from each of the three methods were trained using logistic regression and random forest to predict the phenotypes of unseen samples through 10-fold cross-validation. The area under the ROC curve (AUC-ROC) was used to evaluate the classification accuracy of both methods. Aside from the original dataset used to assess the power of the methods, we also created three types of null scenarios using different permutation strategies (for details see Methods). On the original dataset, MUSTARD consistently outperforms Pseudobulk-PCA and MOFAcell in out-of-sample predictions, achieving a high AUC (Fig. 1f), while Pseudobulk-PCA and MOFAcell fail to capture the sample-level group differences likely due to its reliance on the average expression levels of each sample without cell-level or trajectory information. Under the null scenarios, both MUSTARD and Pseudobulk-PCA have AUC close to 0.5 as expected, while MOFAcell produced zero sample loadings for all components, making the predictive modeling infeasible. Notably, MUSTARD has AUC around 0.5 when only pseudotime was permuted within each sample while the cell-sample relation was preserved. This result underscores the importance of utilizing pseudotime in capturing sample-level differences. Other methods in Table 1 were not benchmarked against MUSTARD as they do not provide sample-level dimensionality reduction.

Next, we applied MUSTARD to the original COVID-Su dataset (Su et al. 2020), which sequenced the peripheral blood mononuclear cells (PBMCs) from COVID-19 patients with varying symptom severity (28 mild, 27 moderate, and 19 severe) and 15 healthy donors. Since CD8+ T cell activation is a crucial process of the immune response in COVID-19 patients, we constructed a pseudotime trajectory from naive T cells to CD8+ T cells using TSCAN (Ji and Ji 2016), and leveraged them to guide the dimensionality reduction by MUSTARD. The top five MUSTARD components capture major temporal patterns including monotone, flat, and single peak (Fig. 2a). Component 2 and 3 jointly separate samples from different severity levels (*P*-values $=2.04\times 10^{-3},1.78\times 10^{-5}$, respectively, Fig. 2b).

We further examine the top genes (ranked by the absolute value of gene loadings) in the aforementioned components. Component 1 is led by genes monotone to pseudotime (Fig. 2c). Known genes associated with T cell activation, such as *NKG7* and *TCF7*, ranked at the top due to their increasing and decreasing trend along the trajectory, respectively (Fig. 2f-g). Components 2 and 3 are both led by genes differentiating patients with different severity levels (Fig. 2d). For example, four genes (*IFI44*, *OAS2*, *IRF7*, and *MX2*), ranking top in both Component 2 and 3, are upregulated in moderate or severe patients compared to healthy donors or mild patients along the trajectory (Fig. 2f-g), which is consistent with the previous findings indicating CD8+ T cells in moderate patients are programmed to be more terminally differentiated (Su et al. 2020, Mathew et al. 2020). Component 5 is led by genes with a single-peak temporal pattern, including *GZMK* and *JUN* (Fig. 2e-g), which are involved in the transition of cell fate from effector memory T cells to the terminal effector T cell stage (Su et al. 2020, Hou et al. 2023). We also applied the Pseudobulk-PCA method and MOFAcell (Flores et al. 2023) to the COVID-Su dataset, examining both the sample loadings and top-ranked genes in the top five components (Fig. 2, available as supplementary data at *Bioinformatics* online). The results from these two methods show high consistency with each other, as both methods aggregate single-cell data into pseudobulk expression profiles. In contrast, MUSTARD offers a distinct perspective of the data by identifying genes associated with pseudotemporal patterns, with multiple components capturing various dynamic gene expression programs.

**Figure 3:**
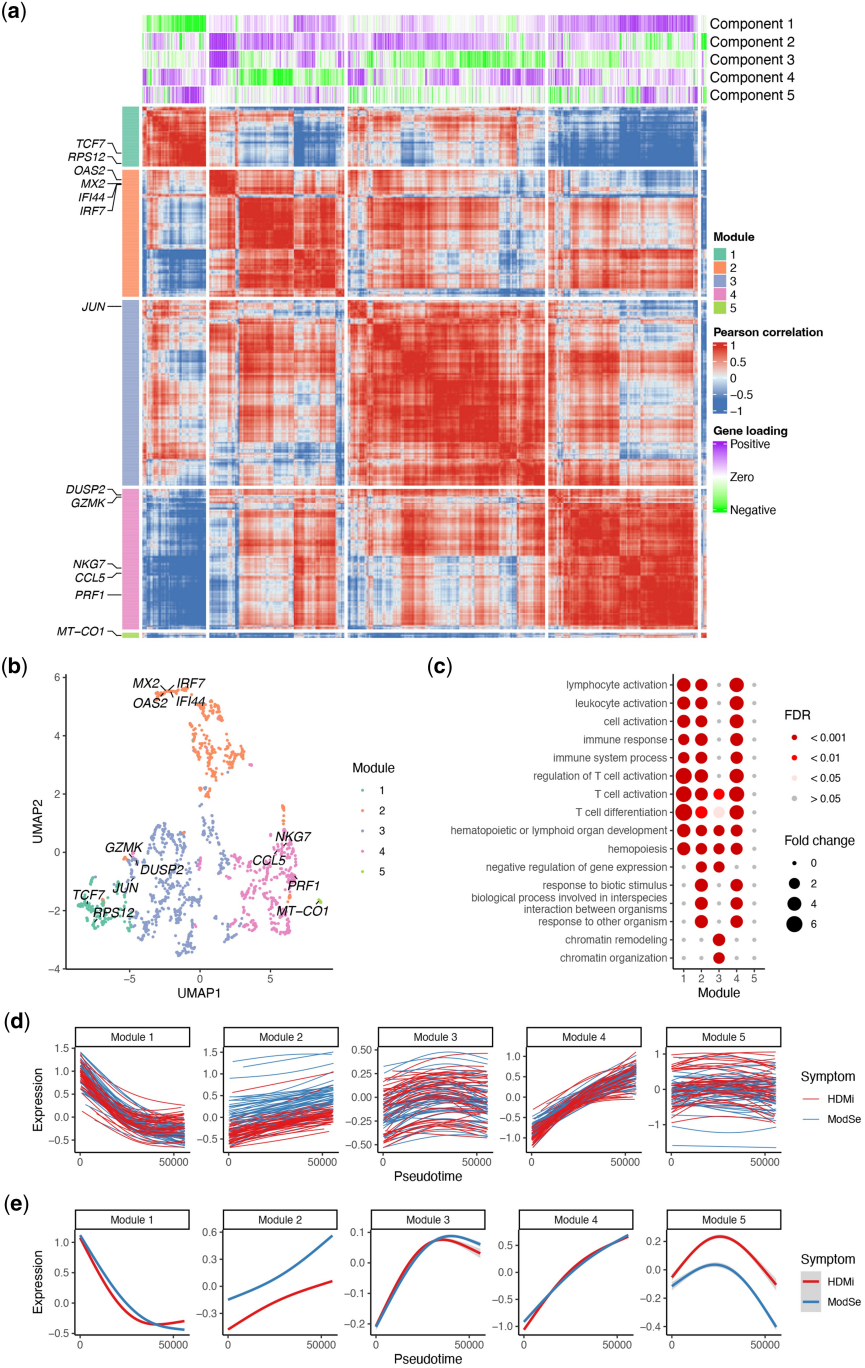
Gene module analysis based on the MUSTARD gene embedding scores for the COVID-Su study. (a) Heatmap showing the gene-gene correlations, determined by gene loadings of the top five components. Example genes in each module are marked. (b) UMAP showing genes from the same MUSTARD module tend to cluster together. (c) Bubble plot showing the enriched gene ontology (GO) terms identified for each gene module, with different modules capturing different aspects of T cell activation. (d-e) Metagenes’ temporal patterns for each sample (d) and each group (e), where Module 2 captures differences associated with symptom severity while Modules 1 and 4 capture monotone trend of genes along the trajectory.

To demonstrate the reproducibility of MUSTARD’s findings from the COVID-Su dataset, we applied it to an independent COVID-19 dataset, referred to as the COVID-Stephenson dataset (Stephenson et al. 2021). This study profiled the PBMCs of 110 individuals (24 healthy, 9 mild, 53 moderate, and 24 severe) from three centers including Ncl, Cambridge, and Sanger. Since MUSTARD is not supervised by any sample-level variables, there is no guaranteed connection between a single component to the phenotype of interest. Consistent with the COVID-Su study, Component 1 captures monotone trend of genes along pseudotime (Fig. 3a, available as supplementary data at *Bioinformatics* online). A closer examination of the leading genes in Component 1 of each study reveals a 75% overlap among their top 100 genes (Fig. 2c and h, Fig. 3c, available as supplementary data at *Bioinformatics* online). Component 4 separates samples from different severity levels (*P*-value $=2.14\times 10^{-5}$), consistent with Components 2 and 3 of the COVID-Su dataset (Fig. 3b, available as supplementary data at *Bioinformatics* online). These components have an overlap of up to 80% among their top 20–100 genes, demonstrating the consistent performance of MUSTARD on the same biological process across different studies (Fig. 2d and h, Fig. 3d, available as supplementary data at *Bioinformatics* online). In contrast, when checking the overlap of components reflecting different aspects of the dataset as a background reference, e.g. Component 2 of the COVID-Su dataset versus Component 1 of the COVID-Stephenson dataset, the percentage of overlap is close to 0 in the leading genes (Fig. 2h).

One notable observation from the MUSTARD analysis of the COVID-Stephenson study is the batch effect captured by Components 2 and 3, demonstrating a clear separation of patients from three different centers (Fig. 2i). Visualizing cell-level low-dimensional representations (e.g. UMAP) is commonly used as a preliminary step to determine whether batch effects exist in a dataset. However, cell-level visualization often becomes less informative as the number of cells reaches millions, with differences between cell types frequently overshadowing sample-level heterogeneity (Fig. 3e, available as supplementary data at *Bioinformatics* online). MUSTARD simplifies the visualization of batch effects from large multi-sample scRNA-seq data by capturing such effects at the sample level, reducing the complexity from millions of cells to hundreds of samples. Additionally, by investigating the leading genes in these components that exhibit the most divergence across three sites, MUSTARD elucidates the underlying factors contributing to the batch effect and offers a strategy for minimizing batch effects during the sequencing step (Fig. 3f-h, available as supplementary data at *Bioinformatics* online). As a pioneering approach to sample-level dimensionality reduction, MUSTARD simultaneously captures both technical effects and biological signals, making it a potent tool for uncovering previously unrecognized sample heterogeneity and paving the way for groundbreaking scientific discoveries.

In addition, the gene loadings obtained from MUSTARD can be used to construct pseudotime-informed gene modules. Compared with supervised methods that identify differentially expressed genes one at a time along the trajectory using a spline-based model (Trapnell et al. 2014, Ji and Ji 2016, Qiu et al. 2017, Street et al. 2018, Hou et al. 2023), MUSTARD can use its gene loadings to group genes with similar temporal patterns into modules, effectively aggregating signals from multiple genes in connection with both known and unknown source of heterogeneity, which we demonstrate using the COVID-Su study (Fig. 3). Different from previous methods that identify gene modules from correlation-based co-expression (Langfelder and Horvath 2008, Morabito et al. 2023), MUSTARD builds gene modules (for details see Methods) based on the similarity of their expression patterns along the pseudotime trajectories using gene loadings of top MUSTARD components (3a-b). We evaluated the enrichment of these modules in gene ontology (GO) terms and found common enrichment terms, including immune response and T cell activation, depicted by distinct enrichment patterns across modules (Fig. 3c). The behavior of each gene module along the trajectory can be illustrated through a metagene constructed by aggregating the gene expression in the given module for each sample (Fig. 3d-e, for details see Methods). For example, the metagenes in Module 1 and 4 show a decreasing and increasing trend, respectively, while the metagene in Module 2 differentiate the samples from different severity levels.

**Figure 4:**
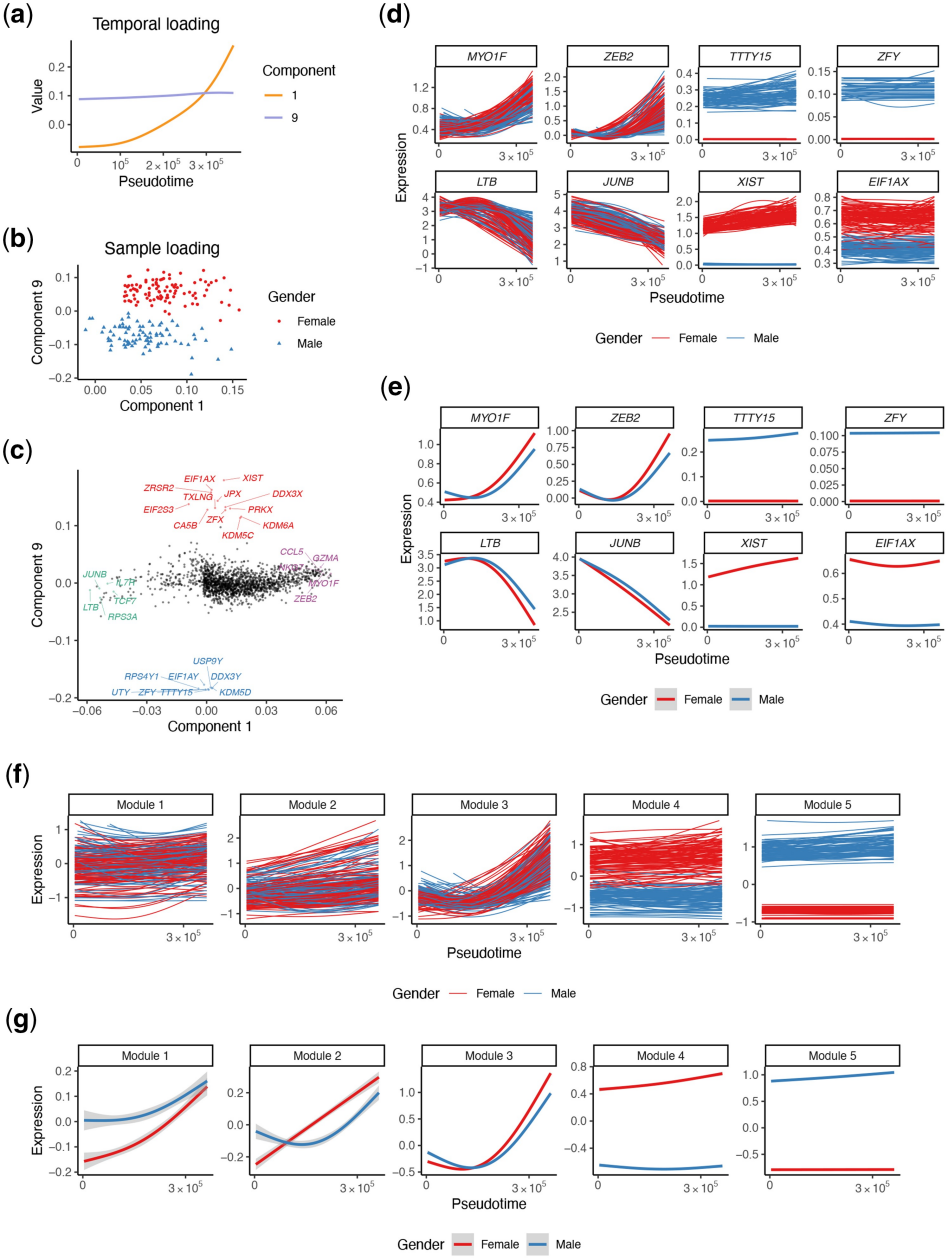
Analysis of TB study. (a-c) Temporal loadings (a), sample loadings (b), and gene loadings (c) in Component 1 and 9. The sex difference was captured by Component 9, whose top genes are all from chromosomes X and Y (colored as red and blue, respectively). Top positive (negative) genes in Component 1 are colored as purple (green). (d-e) Temporal patterns of example genes for each sample (d) and each group (e). (f-g) Temporal patterns of metagenes for modules constructed based on the top 10 components, for each sample (f) and each group (g).

Last but not least, to validate the biological relevance of the genes identified by top MUSTARD components, we applied MUSTARD to a tuberculosis (TB) dataset obtained from a previous study (Nathan et al. 2021), where 337 191 memory T cells from 84 males and 100 females were sequenced. Previous studies have found sex to be a major grouping factor of subjects, which we will use to validate our findings. To demonstrate the flexibility of MUSTARD in utilizing any customized temporal trajectories, instead of inferring pseudotime values using specialized methods, we adopt the trajectory defined in a previous study, which reflects the T cell activation process (Hou et al. 2023). Similar to the results from the aforementioned COVID studies, Component 1 captures the monotone temporal pattern (Fig. 4a), and the top genes in Component 1 show monotone expression patterns along the trajectory (Fig. 4c). Known effector genes, such as *MYO1F* and *ZEB2* increase along the trajectory, while *LTB* and *JUNB* show a decreasing pattern (Fig. 4d-e). The sex difference is captured by Component 9 (Fig. 4b), whose top 20 genes are all from X and Y chromosomes (Fig. 4c-e). In addition, the metagenes constructed from gene module analysis based on the top 10 components reveal several temporal patterns along the trajectory and across samples (Fig. 4f-g).

## Discussion

We present MUSTARD as a pioneering dimensionality reduction tool for multi-sample scRNA-seq data. By leveraging pseudotemporal information, MUSTARD extracts major gene expression variation patterns along pseudotime trajectories and across multiple samples, offering an intuitive sample-level representation of complex multi-sample scRNA-seq data that facilitates the identification of technical batch effects and biologically meaningful heterogeneity, including potential endotypes. MUSTARD effectively links cell-level transcriptomic variation to sample-level heterogeneity and identifies associated gene markers and modules, providing a distinct perspective of the data in the context of psuedotemporal trajectories compared to existing methods, ultimately enhancing our understanding of the relationships between transcriptomic dynamics and sample-level heterogeneity.

It is worth noting that MUSTARD is an unsupervised dimensionality reduction method that does not utilize subject-specific phenotype information in the dimensionality reduction. Therefore, the variation extracted by MUSTARD is not guaranteed to correspond to a known phenotype. Rather, MUSTARD decomposes pre-integration gene expression variation into multiple components, which may reflect both biological signals of interest and technical effects, enabling the detection of previously unknown sample heterogeneity. Users can examine and interpret these components separately, prioritizing components that capture biologically meaningful variation while recognizing components dominated by batch effects.

The dimensionality reduction calculated by MUSTARD is guided by user-provided pseudotime trajectories, providing users with the flexibility to choose which biological process the dimensionality reduction should be focused on. Since MUSTARD assumes the pseudotime to be comparable across samples, misalignment of pseudotime trajectories may result in problematic results from MUSTARD. For the choice of trajectory inference methods, a systematic comparison is beyond the scope of this paper, and we recommend that readers refer to a previous benchmark study for guidance on selecting trajectory inference methods (Saelens et al. 2019). In MUSTARD, TSCAN is used as the default method to computationally infer pseudotime values due to its demonstrated efficacy in a relevant study on pseudotime analysis with multiple scRNA-seq samples (Hou et al. 2023). On the other hand, the flexibility of our method to the pseudotime input also allows it to accommodate broader definitions of trajectories subject to the user, such as any meaningful gradient among the cells.

In our implementation of MUSTARD, we apply a library-size-normalization and log-transformation to the count data, which makes the distribution of observations more Gaussian-like. This allows us to adopt the standard least-squares CP factorization, which implicitly assumes Gaussian noise. While convenient, this assumption is a simplification, as genomic count data are more naturally modeled by Poisson, Zero-Inflated Poisson, or Negative Binomial distributions. Extensions of tensor decomposition that incorporate these distributions, particularly in the Tucker setting, have been studied (Han et al. 2022), and adapting CP factorization on pseudotime scRNA-seq data is an interesting direction for future research.

Selecting the number of components, or the rank, is a critical but challenging aspect of tensor decomposition. Monitoring explained variance or reconstruction error can provide practical guidance, as both quantify how well the model captures variation in the data (see Rank Selection in Supplementary Materials). The explained variance reflects combined effects across samples, genes, and pseudotime. For example, in the TB dataset, sex differences were captured by Component 9 rather than earlier components, since sex-associated genes tend to show weak temporal variation compared to dominant dynamic signals. Therefore, it is often helpful to start with a small number of components and increase the rank until the resulting components correspond to interpretable biological patterns. While a universally optimal procedure for rank selection remains an open question, these practical guidelines balance computational fit with biological interpretability and can support robust applications of MUSTARD. A more systematic approach is left as future work.

MUSTARD, like other methods using optimization, requires the initial values of sample loadings and gene loadings as input. The obtained components with random initializations are highly consistent to those obtained with the original initialization (Fig. 5), suggesting that MUSTARD is robust to different initialization approaches.

MUSTARD is designed for utilizing the pseudotemporal information of one trajectory of interest. When multiple trajectories are considered, the user can apply MUSTARD to each trajectory respectively. Alternatively, the user can concatenate the trajectories. An improvement of MUSTARD for tree-structured pseudotime trajectories will be a focus of our future works.

## Supplementary Material

btag192_Supplementary_Data

## Data Availability

All datasets used in the study are publicly available. The R package MUSTARD with a detailed user manual is publicly available at https://github.com/haotian-zhuang/MUSTARD and Zenodo (DOI: 10.5281/zenodo.18293392) under the MIT license. The source code to reproduce the results in this paper is available at https://github.com/haotian-zhuang/MUSTARD_Paper and Zenodo (DOI: 10.5281/zenodo.18293392) under the MIT license. Fig. 1a was created using BioRender (BioRender.com).
