# Supplementary material for: Trajectory-guided dimensionality reduction for multi-sample single-cell RNA-seq data reveals biologically relevant sample-level heterogeneity
Source: Bioinformatics. 2026 Apr 22;42(5):btag192. doi: 10.1093/bioinformatics/btag192 (PMC13188987; doi:10.1093/bioinformatics/btag192)
Supplement: btag192_Supplementary_Data [file btag192_supplementary_data.pdf]

Supplementary information for "Trajectory-guided dimensionality reduction for multi-sample single-cell RNA-seq data reveals biologically relevant sample-level heterogeneity"

Details of the tensor decomposition

For each component $l = 1, \ldots, r$ sequentially, we perform the following Steps 1 to 3 to estimate $\mathbf{a}^{(l)}$, $\mathbf{b}^{(l)}$, and $\boldsymbol{\xi}^{(l)}$.

Step 1: Initialize $\hat{\mathbf{a}}^{(l)} = (1/\sqrt{m}, \ldots, 1/\sqrt{m})$. Set $\hat{\mathbf{b}}^{(l)}$ as the first left singular vector of mode-2 matricization of $\mathbf{Y}$.

Step 2: Minimize the following function by iteratively updating $\hat{\boldsymbol{\xi}}^{(l)}$, $\hat{\mathbf{a}}^{(l)}$, and $\hat{\mathbf{b}}^{(l)}$ respectively until convergence:

$$\sum_{i=1}^{m}\sum_{j=1}^{n}\sum_{t\in T_i}\left\{y_{ijt} - \eta a_i^{(l)} b_j^{(l)} \xi^{(l)}(t)\right\}^2 + C_K||\xi^{(l)}||^2_{\mathcal{H}}.$$

(2a) Update $\hat{\boldsymbol{\xi}}^{(l)}$ by minimizing

$$\sum_{i=1}^{m}\sum_{j=1}^{n}\sum_{t\in T_i}\left\{y_{ijt} - \hat{a}_i^{(l)} \hat{b}_j^{(l)} \xi^{(l)}(t)\right\}^2 + C_K||\xi^{(l)}||^2_{\mathcal{H}}.$$

with kernel ridge regression. Then normalize $\hat{\boldsymbol{\xi}}^{(l)}$ to $\hat{\boldsymbol{\xi}}^{(l)} = \hat{\boldsymbol{\xi}}^{(l)}/||\hat{\boldsymbol{\xi}}^{(l)}||_2$.

(2b) Update $\hat{\mathbf{a}}^{(l)}$ by

$$\hat{a}_i^{(l)} = \frac{\sum_{j=1}^{n}\sum_{t\in T_i} y_{ijt}\hat{b}_j^{(l)}\hat{\xi}^{(l)}(t)}{\sum_{t\in T_i}[\hat{\xi}^{(l)}(t)]^2}.$$

Then normalize $\hat{\mathbf{a}}^{(l)}$ to $\hat{\mathbf{a}}^{(l)} = \hat{\mathbf{a}}^{(l)}/||\hat{\mathbf{a}}^{(l)}||_2$.

(2c) Update $\hat{\mathbf{b}}^{(l)}$ by

$$\hat{b}_j^{(l)} = \frac{\sum_{i=1}^{m}\sum_{t\in T_i} y_{ijt}\hat{a}_i^{(l)}\hat{\xi}^{(l)}(t)}{\sum_{i=1}^{m}\sum_{t\in T_i}[\hat{a}_i^{(l)}\hat{\xi}^{(l)}(t)]^2}.$$

Then normalize $\hat{\mathbf{b}}^{(l)}$ to $\hat{\mathbf{b}}^{(l)} = \hat{\mathbf{b}}^{(l)}/||\hat{\mathbf{b}}^{(l)}||_2$.

Step 3: Estimate η by minimizing

$$\sum_{i=1}^{m}\sum_{j=1}^{n}\sum_{t\in T_i}\left\{y_{ijt} - \eta \hat{a}_i^{(l)} \hat{b}_j^{(l)} \hat{\xi}^{(l)}(t)\right\}^2$$

with the least squares method. Then update $\mathbf{Y}$ by $y_{ijt} = y_{ijt} - \hat{\eta}\hat{a}_i^{(l)}\hat{b}_j^{(l)}\hat{\xi}^{(l)}(t)$.

Step 4: Estimate $\boldsymbol{\lambda}$ by minimizing

$$\sum_{i=1}^{m}\sum_{j=1}^{n}\sum_{t\in T_i}\left\{y_{ijt} - \sum_{l=1}^{r}\lambda^{(l)}\hat{a}_i^{(l)}\hat{b}_j^{(l)}\hat{\xi}^{(l)}(t)\right\}^2$$

with the least squares method.

The initialization in Step 1 is fixed given the data, and using random initializations does not meaningfully change the results, as shown in Figure S5. Once the initial sample and gene loadings are specified, the algorithm proceeds deterministically, and the estimation of each additional rank does not affect the components obtained in previous steps, thereby improving reproducibility. Although the model formulation and estimation algorithm do not impose orthogonality across components, the components are estimated sequentially, with each fitted component subtracted from the data tensor before the next component is estimated. As a result, later components are encouraged to capture patterns that are not already explained by earlier components. In practice, this leads the components to reflect complementary rather than redundant structure.

Rank Selection

The number of components (i.e., the rank of the decomposition) is a key parameter that directly affects the balance between model complexity and interpretability. Selecting too small a rank may obscure important structure, while choosing too large a rank risks overfitting and generating spurious patterns. In practice, we could apply several computational strategies to guide rank selection, including examining reconstruction error and explained variance.

Reconstruction error and explained variance criteria Set a pre-specified threshold $0 < \tau < 1$. Then choose the smallest r such that the reconstruction error (RE) is no greater than $1 - \tau$, or equivalently, the explained variance (VE) is at least τ:

$$\mathrm{RE}(r) = \frac{\sum_{i=1}^{m}\sum_{j=1}^{n}\sum_{t\in T_i}\left\{y_{ijt} - \sum_{l=1}^{r}\lambda^{(l)}\hat{a}_i^{(l)}\hat{b}_j^{(l)}\hat{\xi}^{(l)}(t)\right\}^2}{\sum_{i=1}^{m}\sum_{j=1}^{n}\sum_{t\in T_i} y_{ijt}^2},$$

$$\mathrm{VE}(r) = \frac{\sum_{i=1}^{m}\sum_{j=1}^{n}\sum_{t\in T_i}\left\{\sum_{l=1}^{r}\lambda^{(l)}\hat{a}_i^{(l)}\hat{b}_j^{(l)}\hat{\xi}^{(l)}(t)\right\}^2}{\sum_{i=1}^{m}\sum_{j=1}^{n}\sum_{t\in T_i} y_{ijt}^2}.$$

These two criteria are equivalent, since $\mathrm{RE}(r) + \mathrm{VE}(r) = 1$.

Moreover, biological validation is also important to the rank selection. One can adopt an iterative approach: start with a small number of components (e.g., 5) and increase the rank until the resulting components correspond to interpretable biological patterns, such as meaningful phenotypes, gene programs, or developmental trajectories. By combining quantitative metrics with domain-specific validation, we can arrive at a rank choice that is both statistically and biologically meaningful.

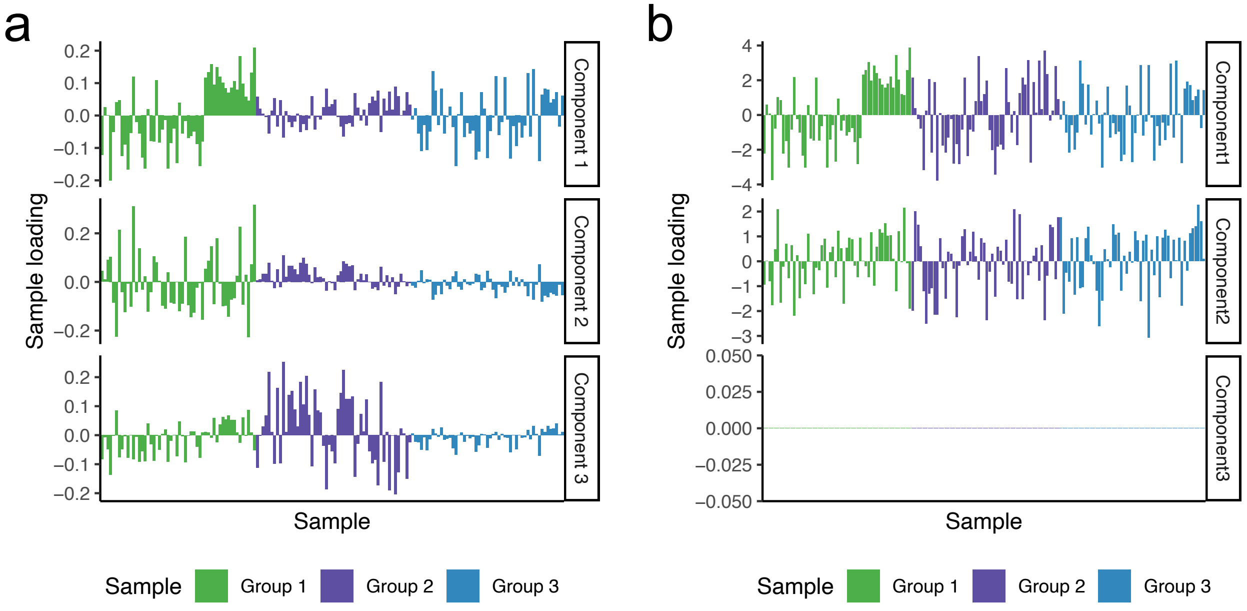

Figure S1: Sample loadings of the top three components identified by Pseudobulk-PCA (**a**) and `MOFAcell` (**b**) in the simulated dataset. They are unable to capture the group differences. Note that `MOFAcell` produced zero loadings for all the samples for the third component.

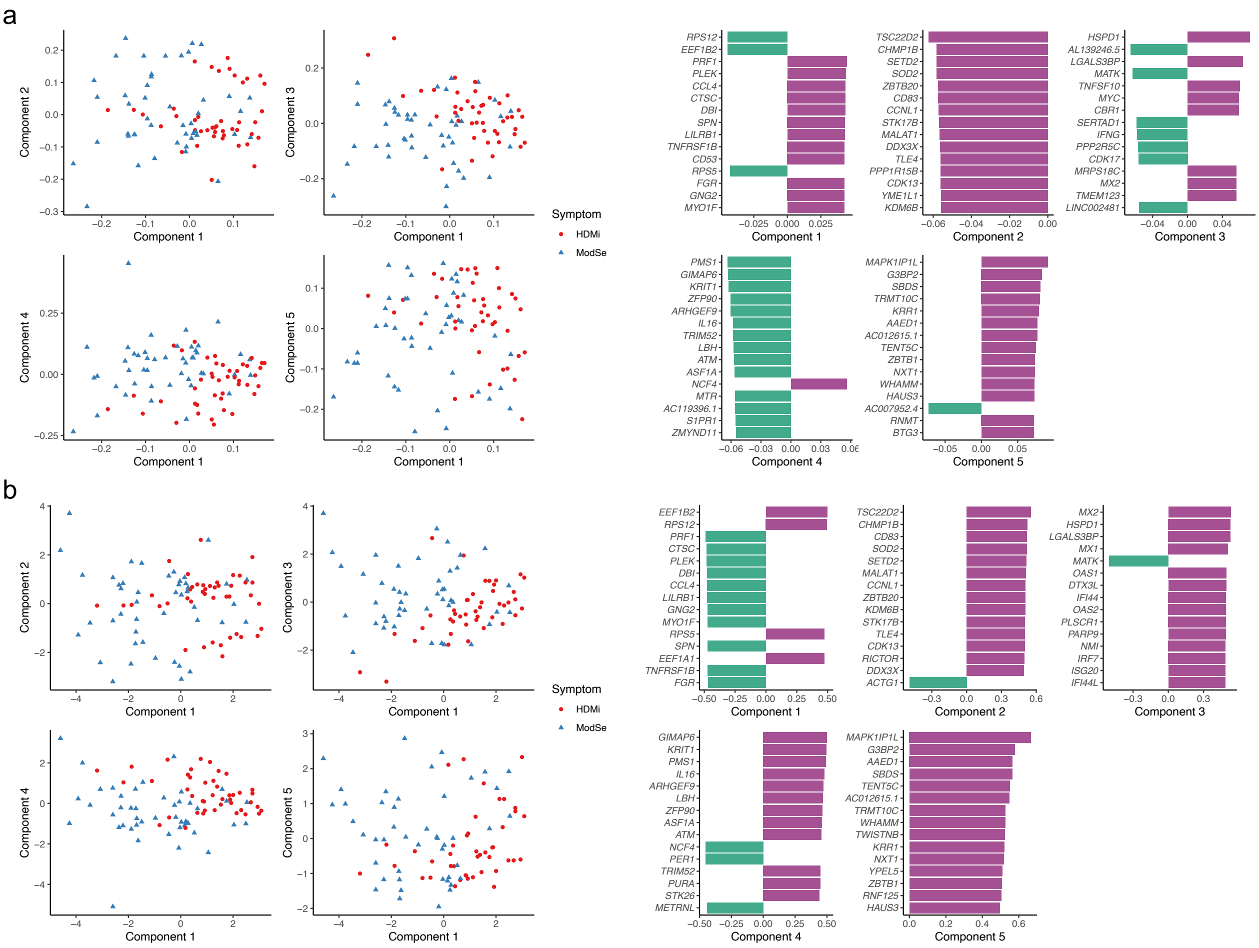

Figure S2: Additional results of COVID-Su study. (**a**), Sample loadings and top genes in the top five Pseudobulk-PCA components. (**b**), Sample loadings and top genes in the top five `MOFAcell` components. Both methods are able to capture differences between symptom severity in top components. The top genes identified by these two methods are similar to each other but different from those identified by `MUSTARD`, demonstrating the unique value of `MUSTARD` in capturing gene expression patterns specific to pseudotemporal trajectories.

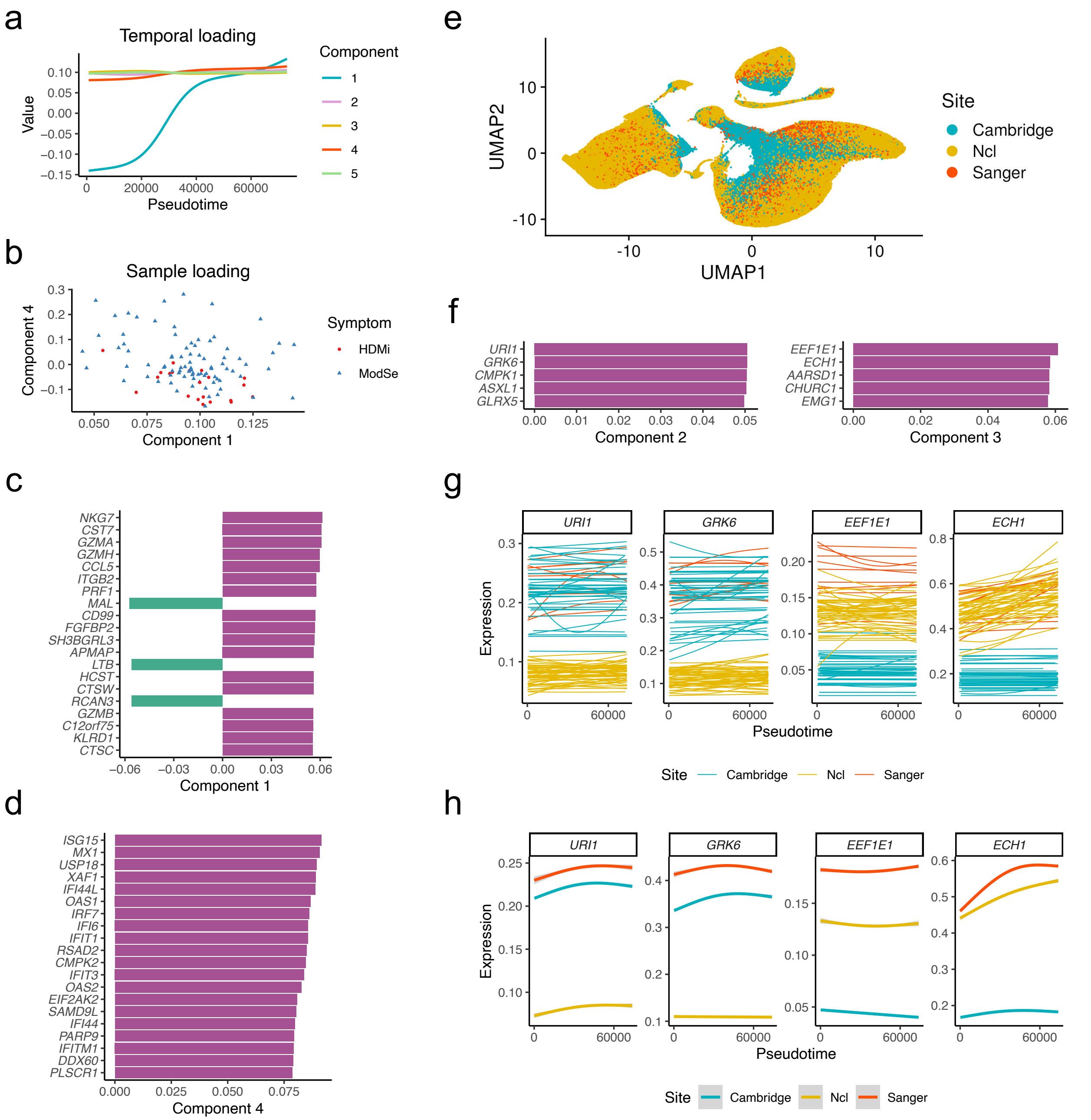

Figure S3: Additional results of COVID-Stephenson study. (**a**), Temporal loadings capture major temporal patterns. (**b**), Component 4 separates samples from different severity levels. p-value obtained by Wilcoxon rank-sum test is 2.14×10^{-5}. (**c-d**), Top genes in Component 1 (**c**) and Component 4 (**d**). (**e**), UMAP with cells colored by three sites. Unlike Figure 2i, here the site differences is overshadowed by cell cluster differences, and the batch effect between Ncl and Sanger is indistinguishable. (**f**), Top genes in Component 2 and 3. (**g-h**), Example genes' temporal patterns for each sample (**g**) and each group (**h**).

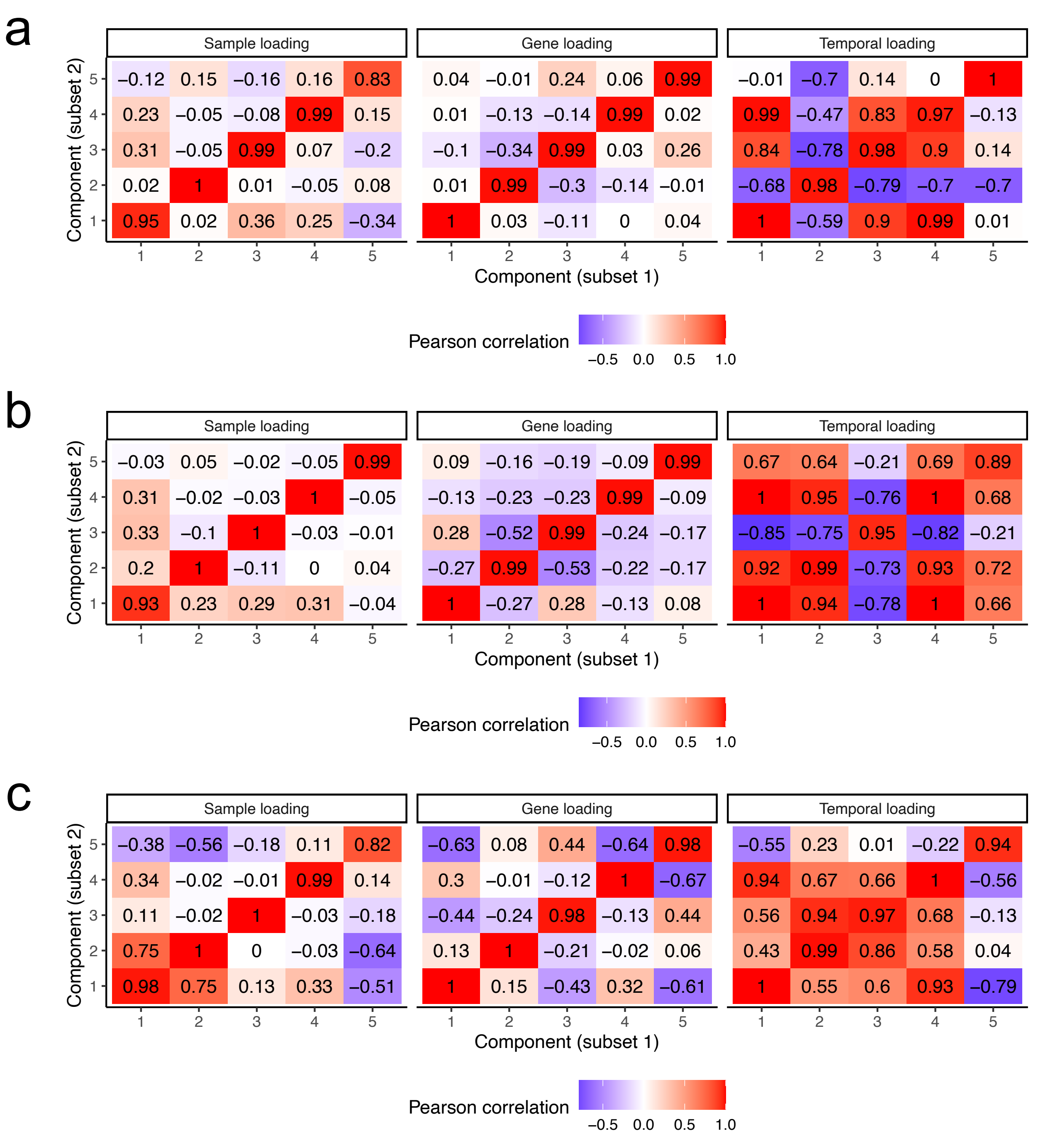

Figure S4: Pearson correlations between sample loadings, gene loadings, and temporal loadings obtained by applying `MUSTARD` on two random subsets of the COVID-Su (**a**), COVID-Stephenson (**b**), and TB datasets (**c**).

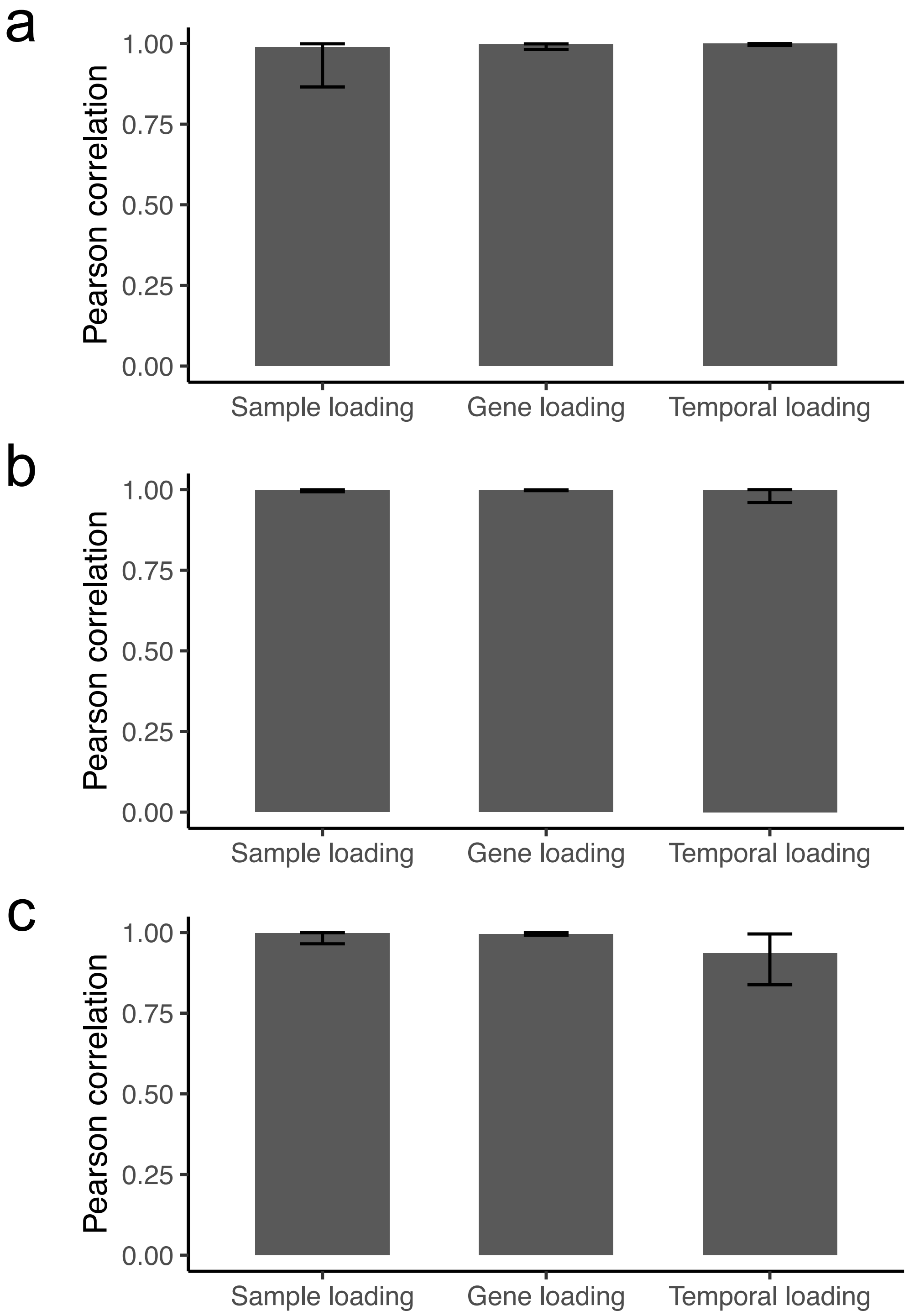

Figure S5: Median Pearson correlations between sample loadings, gene loadings, and temporal loadings obtained by applying `MUSTARD` with the original initialization and 10 random initializations on the COVID-Su (**a**), COVID-Stephenson (**b**), and TB datasets (**c**). The initialization of subject and feature loadings was randomly generated from the standard normal distribution and normalized into norm 1. For each random initialization, each of the top five components from the original initialization was paired with the most highly correlated component (in absolute Pearson correlation) among the top ten components from that random initialization, and median correlations were computed over these pairs for all 10 initializations. Error bars represent the interquartile range (IQR), spanning the 25th to 75th percentiles.
